# Does cognitive impairment influence outcomes from cataract surgery? Results from a 1-year follow-up cohort study

**DOI:** 10.1136/bjophthalmol-2014-305657

**Published:** 2014-10-06

**Authors:** Joanna Mary Jefferis, John-Paul Taylor, Michael Patrick Clarke

**Affiliations:** 1 Clinical Ageing Research Unit, Newcastle University, Newcastle upon Tyne, UK; 2 Institute for Ageing and Health, Newcastle University, Newcastle upon Tyne, UK; 3 Newcastle Eye Centre, Royal Victoria Infirmary, Newcastle upon Tyne, UK

## Abstract

**Background/aims:**

To assess the impact of impaired cognition on visual outcomes 1 year following cataract surgery in a cohort of older people.

**Methods:**

Participants aged 75 years or more with bilateral cataract and scheduled for cataract surgery were recruited consecutively. Cognition was assessed using the revised Addenbrooke's cognitive examination (ACE-R). Participants were divided into two groups: normal (ACE-R ≥88) and impaired cognition (ACE-R <88). Visual quality of life (VQOL) and logarithm of minimum angle of resolution visual acuity (VA) were assessed at baseline and 1 year following cataract surgery.

**Results:**

Of 112 participants, 48 (43%) had normal cognition and 64 (57%) had impaired cognition. One year following cataract surgery participants in both groups had significant improvements in VQOL and VA. Visual outcomes at 1 year were significantly better in participants with normal cognition than in those with impaired cognition (95% CIs for difference 0.4–7.0 and 0.02–0.1, for VQOL and VA, respectively). Regression analyses correcting for potential confounders showed a relationship between baseline cognition and VA at 1 year (R^2^=0.30, p=0.001) and a possible relationship between baseline cognition and VQOL at 1 year (R^2^=0.41, p=0.01, this became insignificant after removal of outliers).

**Conclusions:**

Patients with impaired cognition benefit from cataract surgery, but not to the same extent as patients with normal cognition.

## Introduction

Cataract and cognitive impairment are both age-related health problems which rise in prevalence as populations age. Cataract is the leading diagnosis for ambulatory (outpatient) surgery visits in the USA^1^ and cataract surgery is the most common elective surgical procedure carried out in the UK National Health Service.^2^ The prevalence rate of dementia in people aged 75–79 years is ≈6% and double that in the 80–84 age group.^3^ The prevalence of mild cognitive impairment (MCI) is reported as up to 42% in older populations.^4^ Therefore, in cataract clinics, where many of the patients attending are in older age groups, one would expect to see large numbers of people with dementia and MCI. For example, a cataract surgeon operating on 20 patients a week would be expected to operate on ≈50 patients with dementia each year.^i^ It is, therefore, important to understand the implications of cognitive impairment on visual acuity (VA) and visual quality of life (VQOL) outcomes for people undergoing cataract surgery.

Older age is known to limit improvements from cataract surgery, even when other ocular comorbidities have been taken into account.^5^ ^6^ We have previously hypothesised that this may, in part, be due to cerebral ageing and, therefore, postoperative outcomes may be further limited by the additional presence cognitive impairment.^7^ The aim of this study, therefore, was to assess whether cognitive function influenced visual outcomes from cataract surgery in a cohort of older people.

## Methods

This research adhered to the tenets of the declaration of Helsinki. Ethics committee approval was obtained from the County Durham and Tees Valley research ethics committee.

### Participants

Participants were recruited consecutively from preassessment cataract clinics at a single study centre in the North East of England between March 2011 and August 2012. Participants were eligible for inclusion if they were aged 75 years or more, had bilateral cataract, were scheduled for first eye cataract surgery, had no ocular comorbidity, had no visually significant age-related macular changes (assessed using a 78D lens at the slit lamp and compared with standardised photographs based on the guidelines laid out in^8^), were fluent in the English language, had a mini-mental state examination (MMSE) score >12 and had capacity to consent to participation. Informed consent was obtained from all participants.

### Selection of a patient reported outcome measure

Careful consideration was given to the selection of a suitable patient-reported outcome measure (PROM) for the current study. Guidance was taken from the Cochrane Health Related Quality of Life methods group^9^ and previous reviews of the use of PROMs in ophthalmology.^10^ ^11^ The National Eye Institute 25 item Visual Functioning Questionnaire (VFQ-25)^12^ was selected for its favourable psychometric properties, its short administration time (10 min) and the breadth of concepts covered (including those about visual symptoms and functioning as well as the mental health and social influences of vision). The VFQ-25 has also been used widely in previously published literature and in a variety of different eye diseases (it is not specific to cataract). The VFQ-25 is scored from 0 (worst VQOL) to 100 (best VQOL).

### Assessment measures

Participants were assessed preoperatively and at 1 year following first surgery. An interim appointment was also made at 3 months postoperatively if having single eye surgery or 2 months after second eye surgery if having sequential eye surgeries. Baseline assessment included demographic and medical questionnaire, clinical history from the participant and where available an informant detailing any cognitive symptoms, grading of lens opacities with the lens opacity classification system III (LOCSIII)^13^ and age-related macular disease (AMD) grading based on the guidelines of the international classification and grading system.^8^ At baseline, interim appointment and 1 year postoperatively the following assessments were performed: logarithm of minimum angle of resolution (logMAR) VA, Addenbrooke's Cognitive Examination (ACE-R; which includes the MMSE),^14^ National Eye Institute VFQ-25^12^ and 15 item Geriatric Depression Scale (GDS-15).^15^ VA was defined as best logMAR VA in the better eye, corrected with up to date refraction and/or pinhole. The ACE-R is scored from 0 (worst cognition) to 100 (best cognition). The LOCSIII system for grading cataract scores four different aspects of lens opacity (nuclear colour, nuclear opalescence, cortical and posterior subcapsular) from 1 (no opacity) to 5 or 6 (most opacity). As a summary for cataract grade, we took the highest of these four scores for each eye and then used the grade from the eye with the least cataract (lower grade). AMD was graded as 0 (no changes), 1 (insignificant changes) or 2 (mild changes), and the grade from the least affected eye was used for analysis.

Participants were classified according to the predefined cut-offs on the ACE-R into normal cognition (ACE-R ≥88) and impaired cognition (ACE-R <88). This cut-off has been reported to have a sensitivity of 0.94 and a specificity of 0.89 for detecting dementia.^14^ To aid clinical transferability of the results participants were also grouped according to whether they met clinical criteria for dementia or MCI. History taking and cognitive assessment from the participant and, where possible, an informant was taken by a clinician (JMJ). The diagnostic criteria for dementia from the Diagnostic and Statistical Manual V.4 were used to determine whether participants met the criteria for dementia^16^ and Petersen criteria were used to determine whether participants had MCI.^17^ Any dementia/MCI cases and borderline cases were discussed with an expert clinician (J-PT) to confirm or refute the diagnosis. To mimic clinical practice, no absolute cut-off values were used to define either dementia or MCI, but a whole clinical picture taking into account: the participant's level of functioning, ACE-R score, level of education and any report from participant, informant or another clinician that cognitive performance had changed from a previous level.

### Statistical analysis

Statistical Package for Social Scientists (SPSS) V.17 was used for all the statistical analysis. p Values <0.05 were considered significant. Variables of interest were compared between cognitive groups and between those completing and not completing 1-year follow-up. Variables were compared using Pearson χ^2^ test (dichotomous variables), independent t test (normally distributed variables) or Mann-Whitney U test (non-normally distributed variables).

Comparisons of visual measures (VQOL or VA) at baseline versus 1 year were made with paired t tests. Where significant differences were found, paired t tests were used to compare 3-month postop measures to baseline and 1-year measures. Between-group comparisons were made with independent t tests.

Analysis of covariance (ANCOVA) was used to compare outcome (VQOL or VA) at 1 year between cognitive groups while controlling for that measure at baseline. This approach was chosen as it generally gives greater statistical power than using the change from baseline or the percentage change as the outcome variable. The assumption of homogeneity of variance was checked using variance ratios compared against critical values for Hatley's F_max_.^18^

Linear regression models were used to assess the relationship between baseline cognition (predictor variable) and visual outcomes at 1 year (outcome variable) while correcting for potential co-predictors. The visual outcomes VQOL and VA were analysed separately and corresponding measures at baseline were entered as co-predictors. Other potential predictors were selected a priori if known to affect VA, VQOL and/or cognition as: age, gender, education, AMD grade, cataract grade, GDS-15 score and unilateral/sequential surgery. A backwards stepwise regression model was used to eliminate statistically redundant co-predictors. Multicollinearity and auto-correlation were checked for using tolerance statistics and Durbin–Watson test statistics, respectively. A normal probability plot of standardised residuals was used to ensure they were normally distributed. Individual cases with standardised residuals outside the range ±3.0 were identified and the regression model repeated excluding them.

The main analysis was based on a complete case analysis. To ensure losses to follow-up did not affect significantly the results, we also performed last outcome carried forward analyses where appropriate.

## Results

**Figure 1:**
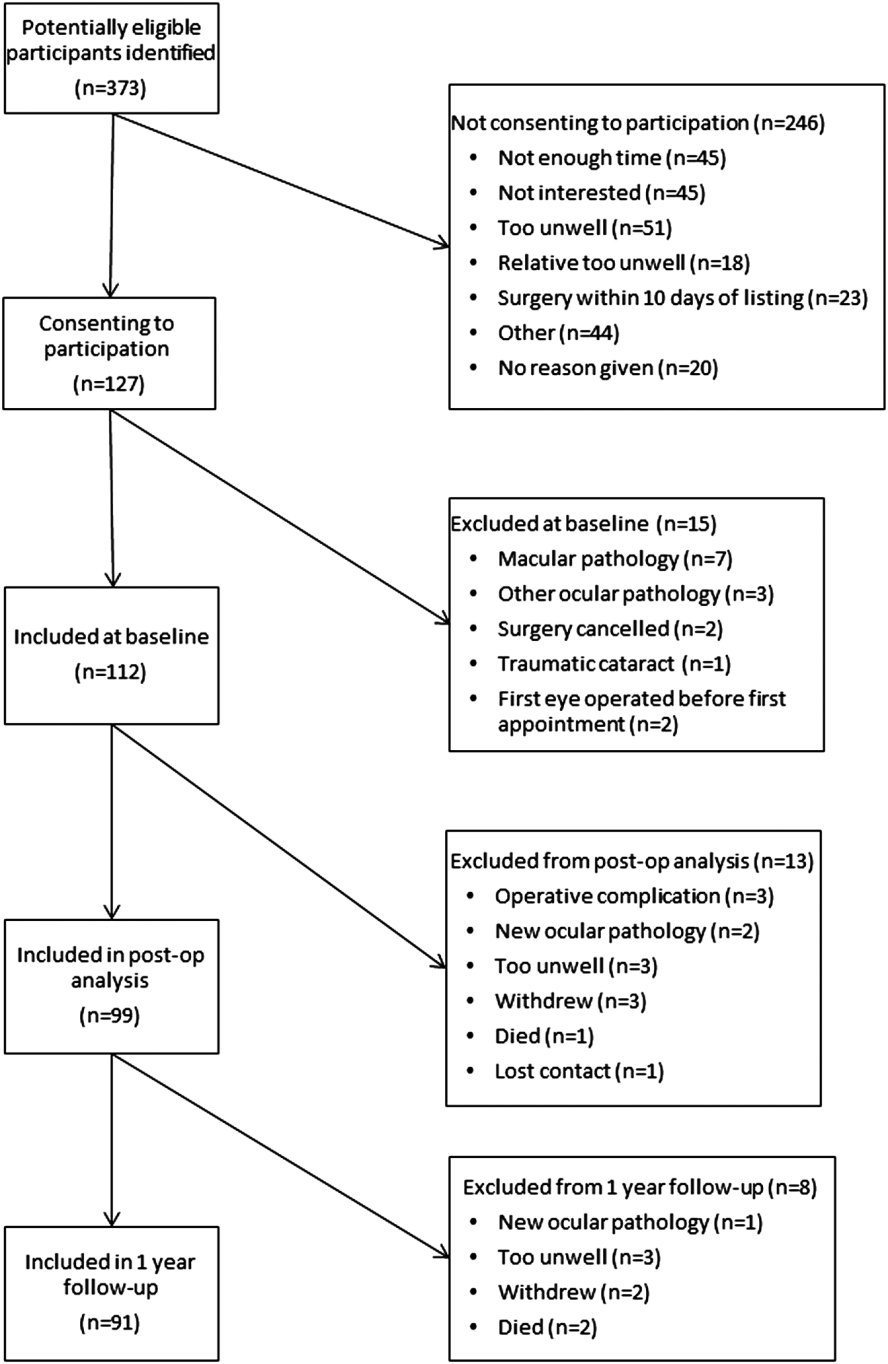
Recruitment and Retention flow- chart.

Figure 1 shows a summary of recruitment and follow-up numbers. A total of 112 participants were included in the baseline analysis, 99 (88%) at the interim appointment and 91 (81%) at 1 year. Of the 112 participants at baseline, 64 (57%) had an ACE-R score <88 and were classified as having impaired cognition. For all participants, baseline mean ACE-R score (SD) was 83.7 (10.3). Of the three participants who died during the study follow-up (figure 1) all were in the impaired cognition group. There were significantly more participants completing follow-up in the normal cognition group (96%) than in the impaired cognition group (70%; Pearson χ^2^, p=0.001). Table 1 shows the baseline characteristics for participants and a comparison between cognitive groups.

**Table 1:** Demographics of participants at baseline comparing cognitive groups

	Cognitive group		
	Normal (n=48)	Impaired (n=64)	p Value*
Age, mean (SD), years	80.0 (3.8)	81.2 (3.9)	0.11
Gender, n (%) men	20 (42)	30 (47)	0.58
Years full-time education, median (IQR)	11 (10–13)	10 (9–11)	<0.001
Depression score, GDS-15, median (IQR)	2 (1–3.75)	3 (1–5)	0.03
Cognition score, ACE-R, mean (SD)	92.3 (2.9)	77.2 (9.0)	<0.001
VFQ-25, mean (SD)	79.9 (12.5)	77.3 (14.9)	0.34
logMAR VA, mean (SD)	0.13 (0.09)	0.21 (0.17)	0.002
AMD grade, median (IQR)	0 (0–1)	0 (0–0)	0.45
Cataract grade, mean (SD)	3.48 (0.54)	3.60 (0.69)	0.33

*p Value refers to independent t test, Pearson-χ^2^ or Mann–Whitney U as appropriate. Note, that higher GDS-15 scores represent poorer mood, higher ACE-R scores represent higher cognition, higher VFQ-25 scores represent better functioning, higher logMAR scores represent worse VA, higher AMD grade indicates more macular changes and higher cataract grade represents more dense cataract.

ACE-R, Addenbrooke's Cognitive Examination; AMD, age-related macular degeneration; GDS-15, 15 item Geriatric Depression Score; logMAR, logarithm of minimum angle of resolution; VA, visual acuity; VFQ-25,25-item Visual Functioning Questionnaire.

Of the 91 participants completing follow-up, 68 (74.7%) had undergone bilateral sequential surgeries. There were no significant differences in the number of participants who had undergone bilateral surgeries between the impaired cognition and normal cognition groups (Pearson χ^2^, p=0.76).

A total of nine (8.0%) participants met a diagnosis of dementia and 23 (20.5%) had MCI. Of these 32 participants with either dementia or MCI, only three (9%) had previously been seen in a memory clinic.

Table 2 compares visual measures (VA and VQOL) between baseline and 1 year for the two cognitive groups. It also shows comparisons of VA and VQOL between cognitive groups. Significant differences between the two cognitive groups for VQOL (F=4.8, p=0.03) and VA (F=4.9, p=0.03) remained robust after the application of an ANCOVA with VQOL/VA at 1 year the dependent variable and VQOL/VA at baseline a covariate. Whole group analysis showed that participants (n=91) had improved VQOL at 1 year compared with baseline (mean difference 13.5, 95% CI 11.0 to 15.9) and the same was true for VA (0.12, 0.10 to 0.15). Interim analysis showed that VQOL improved at interim appointment compared with baseline (mean difference 11.9, 95% CI 9.6 to 14.2) and further improved at 1 year compared with interim (1.5, 0.0 to 3.1). VA also improved at interim appointment compared with baseline (mean difference 0.12, 95% CI 0.09 to 0.14) but not further at 1 year compared with interim (0.00, −0.01 to 0.02).

**Table 2:** Visual outcomes at baseline and 1 year for the two cognitive groups

	Baseline mean (SD)	1 year mean (SD)	95% CI for difference between baseline and 1 year*	p Value*
Outcome: visual quality of life				
Normal cognition (n=46)	79.3 (12.4)	93.7 (5.5)	11.2 to 17.7	<0.001
Impaired cognition (n=45)	77.6 (13.9)	90.0 (9.6)	8.6 to 16.3	<0.001
95% CI for difference between cognitive groups†	−3.8 to 7.8	0.4 to 7.0		
p Value†	0.5	0.03		
Outcome: best corrected visual acuity				
Normal cognition (n=46)	0.13 (0.09)	0.00 (0.09)	0.09 to 0.16	<0.001
Impaired cognition (n=45)	0.18 (0.14)	0.06 (0.11)	0.08 to 0.16	<0.001
95% CI for difference between cognitive groups†	0.00 to 0.10	0.02 to 0.10		
p Value†	0.05	0.007		

*From paired t tests.

†From independent t tests.

When missing data at 1 year follow-up was imputed using a last outcome carried forward approach, paired t tests comparing 1-year follow-up with baseline for the 48 participants with normal cognition showed improved VQOL (mean difference 13.8, 95% CI 10.5 to 17.0) and improved VA (0.12, 0.09 to 0.16). Similarly, for the 64 participants with impaired cognition, comparisons between 1 year and baseline showed improvements in VQOL (mean difference 9.5, 95% CI 6.4 to 12.6) and VA (0.11, 0.07 to 0.15).

When participants were grouped according to the clinical diagnosis, those with dementia or MCI saw significant improvements in VQOL (mean difference 10.2, 95% CI 2.8 to 17.5) and VA (0.1, 0.03 to 0.16). When compared with those not reaching a diagnosis of dementia or MCI, the dementia/MCI group had significantly poorer VQOL at 1 year (mean difference 5.2, 95% CI 1.4 to 9.0) and significantly poorer VA at 1 year (0.07, 0.02 to 0.12).

Table 3 shows the results of the regression analyses. For each visual outcome, a summary of the initial model (including all predictors) and the final model (after backwards stepwise regression removed statistically redundant predictors) is shown. Cognition was a significant predictor of VQOL in the model but this became insignificant following the removal of outliers with absolute standardised residuals >3. Therefore, there remains uncertainty as to whether cognition was a significant predictor of VQOL. Cognition was, however, a significant predictor of VA, both in the initial and final regression models, with higher ACE-R score (better cognition) predictive of lower logMAR VA score (better vision; table 3).

**Table 3:** Backwards stepwise regressions examining relationship between baseline cognition and visual outcomes while correcting for potential confounders

Outcome: visual quality of life at 1 year				
*Predictors entered into initial model:* baseline VFQ-25, age, gender, education, AMD grade, cataract grade, unilateral/sequential surgery, GDS at 1 year, ACE-R				
Initial model:	n=91, R^2^=0.44, ANOVA for model: F=6.7, p<0.001 β for ACE-R=0.20, p=0.03			
Final model:	n=91, R^2^=0.41, ANOVA for model: F=19.1, p<0.001			

	**B**	**SE(B)**	**β**	**p Value**

Constant	66.59	8.42		<0.001
Baseline VFQ-25	0.15	0.06	0.25	0.009
GDS	−1.18	0.30	−0.39	<0.001
ACE-R	0.19	0.08	0.22	0.014†
Outcome: visual acuity at 1 year				
*Predictors entered into initial model:* baseline VA, age, gender, education, AMD grade, cataract grade, unilateral/sequential surgery, GDS at 1 year, ACE-R				
Initial model:	n=91, R^2^=0.30, ANOVA for model: F=3.9, p=0.001 β for ACE-R=−0.30, p=0.005			
Final model:	n=91, R^2^=0.28, ANOVA for model: F=11.2, p<0.001*			

	**B**	**SE(B)**	**β**	**p Value**

Constant	0.37	0.10		<0.001
Baseline VA	0.30	0.08	0.32	0.001
Unilateral/sequential surgery	−0.05	0.02	−0.22	0.02
ACE-R	−0.003	0.001	−0.32	0.001

*When the two cases with standardised residuals >3 were removed, ACE-R had a p value of 0.5 in the initial model and was removed by the stepwise regression before the final model.

ACE-R, Revised Addenbrooke's Cognitive Examination; AMD, age related macular degeneration; GDS, Geriatric Depression Scale; VA, visual acuity; VFQ-25, 25 item Visual Functioning Questionnaire; ANOVA, analysis of variance.

## Discussion

In this group of older people attending for cataract surgery from the community, 64 (57%) had ACE-R scores <88 and 32 (29%) met the diagnostic criteria for dementia or MCI, suggesting that there are significant levels of cognitive impairment in a typical UK-based cataract clinic. Measures of VQOL and VA improved significantly for participants across all levels of cognition. However, poor cognition adversely affected VA outcomes and had a possible impact on VQOL outcomes.

We noted some significant differences in demographics between the two cognitive groups (see table 1), with lower levels of education in the impaired cognition group. This is not unexpected, as education and with it, poorer socioeconomic status is a known risk factor for cognitive impairment.^19^ Furthermore, those with lower educational level may struggle with certain aspects of cognitive assessment. We also saw lower mood in the cognitively impaired group. Poor mood may impair test performance on cognitive tasks due to poor concentration and motivation; depression is known to be associated with dementia and may be an independent risk factor for developing dementia.^20^ There was a difference in baseline VA between the two cognitive groups (table 1) but no differences in cataract density or macular changes. This supports the idea of a more direct relationship between vision and cognition, not simply as a result of higher levels of recognisable ocular comorbidity in those with lower cognition.

For participants in both groups, we saw a significant benefit following cataract surgery in terms of VQOL and VA. The mean improvements in VQOL of 12.4 (impaired cognition group) and 14.4 (normal cognition group) (see table 2) are clinically significant and similar to improvements reported previously following cataract surgery.^21^ ^22^ The improvement in VA of 0.12 is equivalent to six letters (or just over one line) on the logMAR VA chart; this was significantly less than reported improvements in visual acuities seen in a recent UK audit,^23^ but this looked at improved vision for individual eyes (monocular vision) and not for patients (binocular vision) as we have done here. These results highlight the use of cataract surgery for patients in both normal and cognitively impaired populations.

Despite both groups seeing improvements in visual outcomes, there were significant differences between the groups in terms of VQOL and VA outcomes at 1 year (table 2). These differences remained when using an ANCOVA to correct for baseline VA/VQOL measures. Similar results were also seen when participants were grouped according to diagnosis (MCI or dementia) as opposed to cognitive score. While differences between impaired cognition and normal cognition groups in terms of VA and VQOL at 1 year were statistically significant, they were somewhat small and may not be clinically significant (table 2).

As well as considering participants divided into cognitive groups, we have examined cognition as a continuous variable in the regression analysis. While the dichotomisation of ACE-R scores is useful for clinical and statistical interpretation, studying cognition as a continuous variable affords greater statistical power.^24^ Regression analysis allows correction for potential confounders (eg, education/comorbid depression) and these analyses (table 3) confirmed a significant association between cognition and VA. The relationship between cognition and VQOL was less significant and not robust to removal of outliers. The association between cognition and vision cannot easily be explained by macular changes or denser cataract in those with cognitive impairment as there were no differences between cognitive groups in cataract or AMD measures (table 1), and they were corrected for in the regression model (table 3). Other possible explanations include higher levels of unrecognised pathology in those with lower cognition (eg, retinal or optic nerve pathology not identifiable by slit lamp biomicroscopy)^25^ ^26^; a common underlying degenerative aetiology for vision and cognitive impairment^7^ or an impact of cognition on attention, decision making and judgement skills required to read down a letter chart.^27^ These factors could also account for the differences in VA seen between cognitive groups at baseline (see table 1).

In this sample of older people, 57% of participants fell below the ACE-R cut-off of 88 points, a significantly higher percentage than those meeting a diagnosis of dementia or MCI (29%). It has been suggested that an ACE-R cut-off of <88 may be too stringent for day-to-day clinical practice and hence overestimate dementia,^28^ and this study would support this assertion. Our cohort was older than those used to describe these cognitive cut-offs and there is a lack of normative data on the ACE-R for people aged over 75 years.^14^ Our data does, however, suggest that there are significant levels of undiagnosed dementia within the eye clinic and thus ophthalmologists may have a role in initiating onward referrals.

Our recruitment and attrition rates (figure 1) are in line with other studies in similar populations.^29^ ^30^ However, we saw significantly higher levels of attrition in the impaired cognition group and this may limit the generalisability of the results. To address the possibility that losses to follow-up were not completely at random (people with poor cognition or poor vision may find it harder to complete the study follow-up), we used a last outcome carried forward analysis which did not alter our conclusions. However, in this study, we have not considered those with severe cognitive impairment and thus the conclusions cannot necessarily be extrapolated to this patient group.

This research emphasises the use of cataract surgery in those with both normal and impaired cognition, with both groups experiencing significant improvements in visual outcomes. Cognitive impairment may, however, limit visual improvements following cataract surgery. Ophthalmologists helping patients to make decisions about whether to undergo cataract surgery need to take into account cognitive impairment, in the same way that older age and ocular comorbidities are considered as factors that may limit visual outcomes from cataract surgery. Meanwhile, further research is needed to understand the relationship between vision and cognition we have noted. It could be due to poor attention and concentration during VA testing in those with reduced cognition, as opposed to truly poorer vision suggesting that different approaches to accurately assess VA in patients with suspected cognitive impairment are needed.

## Footnotes

i Assumes 20% of patients from each of the age groups: 60–64, 65–69, 70–74, 75–79 and 80–84 years with % prevalence rates for dementia of 0.9, 1.5, 3.6, 6.0 and 12.2, respectively.^3^
